# Paraventricular, subparaventricular and periventricular hypothalamic IRS4-expressing neurons are required for normal energy balance

**DOI:** 10.1038/s41598-020-62468-z

**Published:** 2020-03-26

**Authors:** Ames K. Sutton Hickey, Ian E. Gonzalez, Marianna Sadagurski, Michael Rajala, Chunxia Lu, Margaret B. Allison, Jessica M. Adams, Martin G. Myers, Morris F. White, David P. Olson

**Affiliations:** 1 Department of Molecular and Integrative Physiology, University of Michigan, Ann Arbor, MI USA; 2 Department of Internal Medicine, University of Michigan, Ann Arbor, MI USA; 3 Department of Endocrinology, Children’s Hospital Boston, Boston, MA USA; 4 Division of Pediatric Endocrinology, Department of Pediatrics, Michigan Medicine, Ann Arbor, MI USA

**Keywords:** Hypothalamus, Homeostasis

## Abstract

Understanding the neural components modulating feeding-related behavior and energy expenditure is crucial to combating obesity and its comorbidities. Neurons within the paraventricular nucleus of the hypothalamus (PVH) are a key component of the satiety response; activation of the PVH decreases feeding and increases energy expenditure, thereby promoting negative energy balance. In contrast, PVH ablation or silencing in both rodents and humans leads to substantial obesity. Recent studies have identified genetically-defined PVH subpopulations that control discrete aspects of energy balance (e.g. oxytocin (OXT), neuronal nitric oxide synthase 1 (NOS1), melanocortin 4-receptor (MC4R), prodynorphin (PDYN)). We previously demonstrated that non-OXT NOS1^PVH^ neurons contribute to PVH-mediated feeding suppression. Here, we identify and characterize a non-OXT, non-NOS1 subpopulation of PVH and peri-PVH neurons expressing insulin-receptor substrate 4 (IRS4^PVH^) involved in energy balance control. Using Cre-dependent viral tools to activate, trace and silence these neurons, we highlight the sufficiency and necessity of IRS4^PVH^ neurons in normal feeding and energy expenditure regulation. Furthermore, we demonstrate that IRS4^PVH^ neurons lie within a complex hypothalamic circuitry that engages distinct hindbrain regions and is innervated by discrete upstream hypothalamic sites. Overall, we reveal a requisite role for IRS4^PVH^ neurons in PVH-mediated energy balance which raises the possibility of developing novel approaches targeting IRS4^PVH^ neurons for anti-obesity therapies.

## Introduction

Genetic polymorphisms associated with obesity are disproportionately clustered in pathways affecting neural function and architecture in the central nervous system (CNS)^1^. Within the brain, the paraventricular nucleus of the hypothalamus (PVH) is a primary hypothalamic node that integrates neural and humoral information regarding energy balance and sends output signals to structures in the hindbrain and spinal cord to control satiety and energy expenditure^2–4^. Dysregulation of the entire PVH with site-directed lesions or inhibition of PVH melanocortin-4 receptor (MC4R) action produce profound obesity^5–7^. However, while disruption of MC4R^PVH^ activity leads to obesity due to hyperphagia, lesions of the entire PVH are associated with both feeding and energy expenditure dysregulation^7–9^. While MC4R^PVH^ activity is necessary and sufficient to alter feeding behavior, MC4R action in the PVH cannot be ascribed to neural populations expressing oxytocin (OXT), vasopressin (AVP), or corticotropin-releasing hormone (CRH)^7^. Manipulation of other PVH populations, such as neuronal nitric oxide synthase 1 (NOS1^PVH^) neurons, is sufficient to alter both feeding and energy expenditure^10^. This raises the possibility that distinct PVH cell types might independently or coordinately regulate feeding and/or energy expenditure. Even though substantial efforts have been made to define PVH neuronal populations and their distinct roles in energy balance control, few studies have revealed PVH populations that are capable of regulating multiple aspects of energy balance (i.e. energy expenditure and feeding), apart from the NOS1^PVH^ population.

*Nos1* expression marks a relatively large percentage of PVH neurons that send projections to hindbrain and spinal cord sites. Chemogenetic activation of NOS1^PVH^ neurons suppresses feeding and increases energy expenditure by promoting both increased physical activity and thermogenesis^10^. OXT^PVH^ neurons are almost entirely contained within the NOS1^PVH^ field, yet their activation drives only small changes in energy expenditure and does not suppress feeding^10^. NOS1^PVH^-dependent changes in energy expenditure are likely independent of MC4R-signaling in the PVH, since activation of MC4R^PVH^ neurons suppresses feeding but does not affect energy expenditure and selective PVH expression of *Mc4R* in an otherwise *Mc4R*-null background has little impact on energy expenditure^8,11^. Given the important contribution of both energy expenditure and feeding dysregulation in the development of obesity, the identification of distinct targets that have the capability to modulate both aspects of energy balance is particularly pertinent.

Recent reports indicate that insulin receptor substrate-4 (IRS4) acts in synergy with insulin receptor substrate-2 (IRS2) in the hypothalamus to maintain normal bodyweight^12^. We discovered that *Irs4* is expressed in and adjacent to the PVH and that the paraventricular and periventricular IRS4-expressing cell population (referred to as IRS4^PVH^) is both necessary and sufficient for normal feeding and bodyweight, suggesting a functional role for PVH and peri-PVH *Irs4-*expressing neurons in the control of energy homeostasis. Additionally, we find that NTS-projecting and PBN-projecting IRS4^PVH^ neurons are densely innervated by local PVH neurons, supporting a role for an intra-PVH network in the regulation of energy balance. Monosynaptic afferent neural tracing suggests that the inputs to IRS4^PVH^ neurons vary depending on the brain regions to which an IRS4^PVH^ subset projects. Overall, our study proposes a novel framework for the regulation of bodyweight consisting of multiple interconnected PVH populations that are potentially under independent control to modulate distinct energy balance parameters including feeding and energy expenditure.

## Results

### Irs4 expression marks a distinct PVH subpopulation

**Figure 1:**
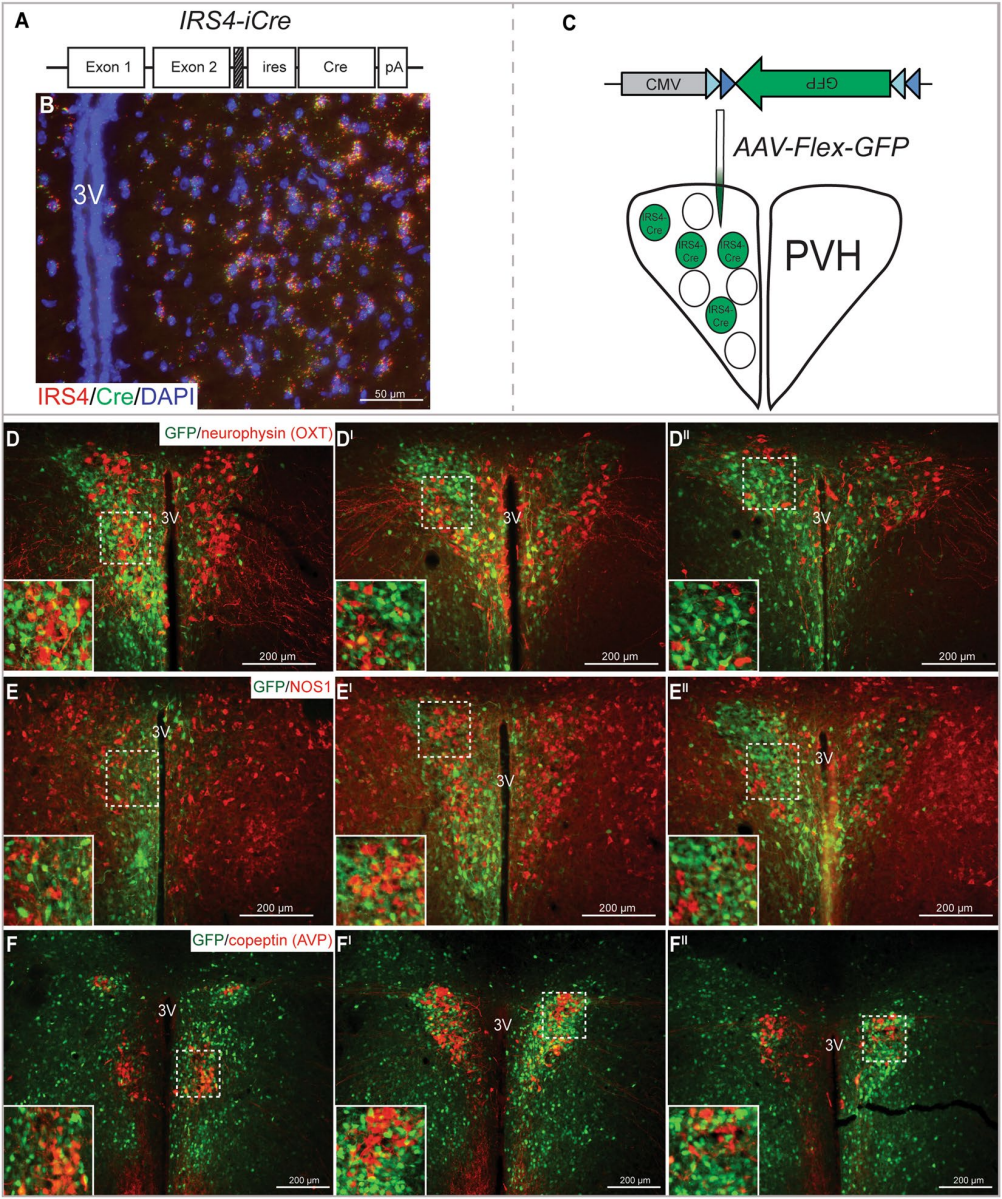
*Irs4* expression defines a PVH subpopulation. *In situ* hybridization was performed on coronal slices from adult *IRS4-iCre* mice (**A**) for *Irs4* (red) and *Cre* (green), showing substantial overlap between *Cre* and *Irs4* mRNA in the PVH (**B**). To identify overlap between IRS4^PVH^ neurons and other PVH populations, mice were stereotaxically injected in the PVH with a Cre-dependent GFP reporter virus (AAV-Flex-GFP, **C**) to visualize Cre-expressing neurons in the adult mouse. Immunohistochemistry (IHC) in the PVH demonstrates limited co-localization between GFP-labeled IRS4^PVH^ neurons (green) and OXT (**D**), NOS1 (**E**), or AVP (**F**) neurons in the PVH. Dashed boxes indicate regions that are digitally enlarged and shown as insets. 3 V = third ventricle.

Hypothalamic IRS4 and IRS2 synergistically contribute to body weight maintenance^12^. Since *in situ* hybridization (Allen Mouse Brain Atlas^13^) reveals dense *Irs4* expression in and adjacent to the PVH, we sought to determine the role of IRS4^PVH^ neurons in energy homeostasis using Cre-dependent technologies in combination with a novel *IRS4-iCre* knock-in mouse model in which Cre recombinase expression is tethered to *Irs4* (Fig. 1A). The *Irs4* gene is located on the X chromosome. Since random X inactivation may lead to variable Cre activity in female mice, male mice were used exclusively in all studies. *In situ* hybridization of both *Cre* and *Irs4* mRNA in the PVH of *IRS4-iCre* mice was used to verify the appropriate expression of Cre recombinase in the *IRS4-iCre* mouse line and demonstrated substantial overlap of *Irs4* and *Cre* mRNA transcripts in the PVH (Fig. 1B). To investigate IRS4^PVH^ neuron overlap with other known PVH populations, IRS4^PVH^ neurons were labeled by injection of a Cre-dependent GFP reporter virus into the PVH of *IRS4-iCre* mice (Fig. 1C). This approach eliminates the possibility of overrepresentation of the IRS4^PVH^ reporter population due to developmental *Irs4* expression and aligns with subsequent Cre-dependent viral technologies used to manipulate IRS4^PVH^ neurons in adult mice. Brains stained for GFP (indicating *IRS4-iCre* activity) and neurophysin I, the carrier protein for oxytocin, indicate that adult IRS4^PVH^ neurons do not substantially overlap with OXT^PVH^ neurons (Fig. 1D). To determine if IRS4^PVH^ neurons are contained within the NOS1^PVH^ population, we stained brain slices from *IRS4-iCre* mice injected with AAV-Flex-GFP for NOS1 peptide and GFP and found that IRS4^PVH^ neurons are a separate population from NOS1^PVH^ cells (Fig. 1E). As AVP^PVH^ neurons are also able to modestly control feeding, we examined the overlap of AVP and IRS4 within the PVH^14^. Indeed, GFP-identified PVH neurons representing *IRS4-iCre* activity do not overlap substantially with immunoreactivity for copeptin, the carrier molecule for AVP (Fig. 1F). To investigate the potential overlap between IRS4^PVH^ neurons and other subpopulations of the PVH recently reported to contribute to energy homeostasis, we conducted *in situ* hybridization for *Irs4* mRNA in combination with *Crh*, *Pdyn*, and thyrotropin-releasing hormone (*Trh*) mRNAs (Supp. Fig. 1.1). The majority of IRS4^PVH^ neurons do not express *Crh* (9.76 + 2.94% of IRS4^PVH^ with *Crh*, n = 3 mice) or *Pdyn* (15.77 + 1.19% of IRS4^PVH^ with *Pdyn*, n = 3 mice), and a modest fraction of CRH^PVH^ and PDYN^PVH^ neurons express *Irs4* mRNA (26.08 + 2.38% and 28.14 + 7.43% respectively). We do find that some IRS4^PVH^ neurons express *Trh* (40.54 + 1.10%), and more than half of the TRH^PVH^ neuronal population expresses *Irs4* mRNA (60.43 + 3.14%). Taken together, these data suggest that *Irs4* expression marks a circumscribed population of PVH neurons defined by the relative absence of OXT, AVP, and NOS1, but some overlap with CRH, PDYN, and TRH.

### IRS4^PVH^ neurons are capable of regulating both feeding and energy expenditure

**Figure 2:**
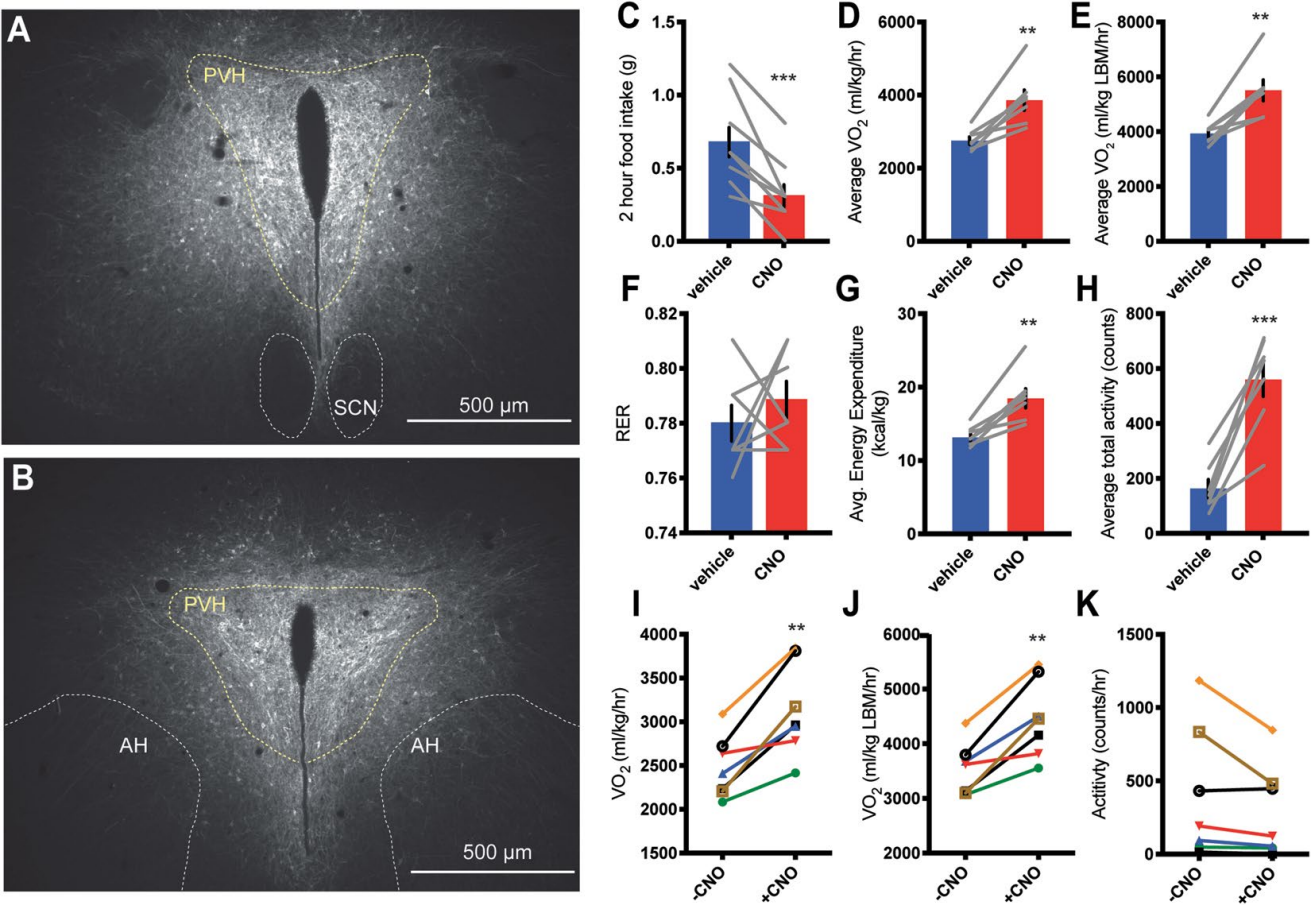
Acute activation of IRS4^PVH^ neurons decreases feeding and increases energy expenditure. IHC for mCherry identifies AAV-hM3Dq expression in IRS4^PVH^ neurons throughout the PVH (**A**,**B**: yellow dotted line). (**C**) Two-hour food intake at the onset of dark following activation of IRS4^PVH^ neurons with an i.p. injection of CNO (0.3 mg/kg) in comparison to vehicle. Activation of IRS4^PVH^ neurons increases total oxygen consumption (**D**,**E**), energy expenditure (**G**), and activity (**H**) over a four-hour time period in the absence of food, whereas respiratory exchange ratio (RER) is not significantly different (**F**). (**I**–**K**) IRS4^PVH^ neuronal activation increases activity-independent changes in oxygen consumption (**I**,**J**), measured at time points four hours before (−CNO) or after (+CNO) activation when activity levels were relatively matched (**K**). Average values ± SEM are shown, significance was determined using a paired-t-test in comparison to vehicle values. **p < 0.01, ***p < 0.001; feeding n = 9, CLAMS n = 7; AH = anterior hypothalamus, SCN = suprachiasmatic nucleus, PVH = paraventricular nucleus of the hypothalamus.

Given the published role of IRS4 in energy homeostasis and its expression in the PVH, we sought to determine the ability of these neurons to regulate distinct aspects of energy balance. To achieve this, we employed Cre-dependent DREADD (Designer Receptors Exclusively Activated by Designer Drugs) viruses to acutely modulate neuronal activity in response to peripheral injection of an otherwise inert compound, clozapine N-oxide (CNO)^15^. To achieve remote activation of IRS4^PVH^ neurons, we performed bilateral PVH injections of AAV-Flex-hM3Dq in *IRS4-iCre* mice (Fig. 2A,B). Following recovery, PVH-directed AAV-Flex-hM3Dq injected *IRS4-iCre* mice were fasted during the day and injected with either vehicle or CNO at the onset of the dark cycle, a time when feeding normally occurs. Activation of IRS4^PVH^ neurons results in robust suppression of feeding (Fig. 2C, p = 0.0005, paired t-test, t = 5.680). Feeding effects are not attributable to CNO or its metabolites as a separate cohort of wildtype mice injected with AAV-Flex-hM3Dq failed to suppress feeding with no change in nuclear Fos expression following injection of CNO (Supp. Fig. 2.1A,B). As pan-activation of PVH neurons suppresses feeding and increases energy expenditure, we placed IRS4^PVH^ mice injected with AAV-Flex-hM3Dq in metabolic chambers to measure energy expenditure and locomotor activity following CNO-mediated activation^10^. In the absence of food, activation of IRS4^PVH^ neurons increases energy expenditure, VO_2_ and total activity without a significant change in RER (Fig. 2D–H; VO_2_: p = 0.0027, t = 4.897; VO_2_ lean body mass (LBM): p = 0.0025, t = 5.001; Energy Expenditure p = 0.0026, t = 4.946; total X activity: p = 0.0007, t = 6.297; all paired t-test). To examine the effect of IRS4^PVH^ activation on resting energy expenditure (REE), we measured VO_2_ at timepoints before and after CNO injection in which physical activity was similar (Fig. 2I–K), as performed previously^10^. This demonstrates that IRS4^PVH^ activation results in increased VO_2_ (normalized to bodyweight or LBM) that is independent of increased physical activity (VO_2_ REE p = 0.0022, t = 5.113; VO_2_/LBM p = 0.0019, t = 5.254). Thus, activation of IRS4^PVH^ neurons suppresses dark-cycle feeding and promotes energy expenditure in the absence of food.

### IRS4^PVH^ neurons project to hindbrain and spinal cord regions

**Figure 3:**
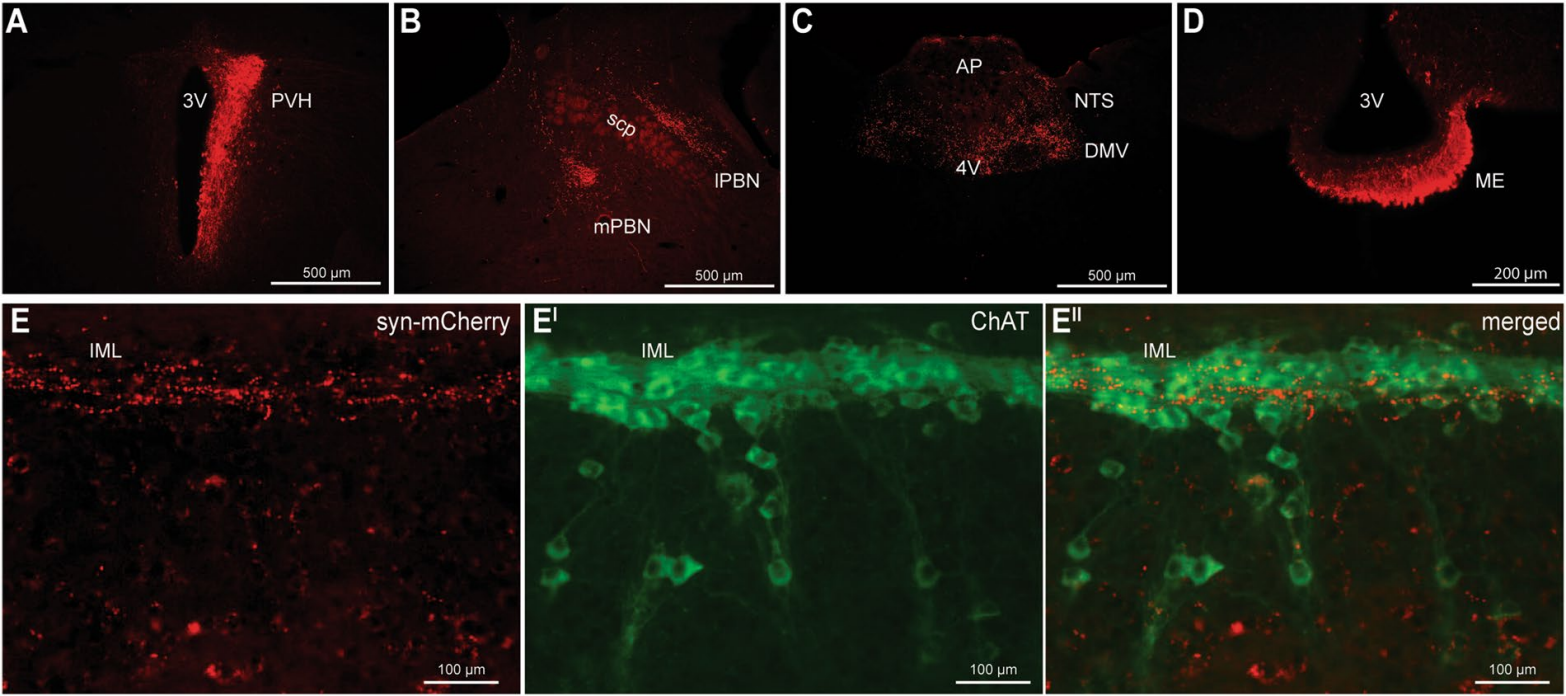
IRS4^PVH^ neurons project to hindbrain and spinal cord regions. (**A**) A Cre-dependent synaptophysin-mCherry adenovirus (syn-mCherry) was unilaterally injected in the PVH area of *IRS4-iCre* mice to trace synaptic terminals throughout the brain. (**B**,**C**) Syn-mCherry positive terminals are observed in hindbrain regions including the medial and lateral parabrachial nucleus (mPBN, lPBN, respectively, (**B**), as well as the dorsal motor nucleus of the vagus (DMV) and nucleus of the solitary tract (NTS, **C**). (**D**) IRS4^PVH^ neurons also project to the median eminence (ME). (**E**) Syn-mCherry (red) identifies synaptic terminals in the intermediolateral column of the spinal cord (IML) in close proximity to neurons expressing choline acetyltransferase (ChAT, green). scp = superior cerebellar peduncle, 4 V = fourth ventricle, 3 V = third ventricle, AP = area postrema.

To identify the neural circuits engaged by IRS4^PVH^ neurons, we used injection of a Cre-dependent adenovirus, synaptophysin-mCherry (syn-mCherry) in the PVH area to label the IRS4^PVH^ synaptic terminals. Unilateral injection of syn-mCherry (Fig. 3A) in the PVH area of *IRS4-iCre* mice identifies robust IRS4^PVH^ projections to hindbrain projection targets including the parabrachial nucleus (PBN, Fig. 3B), nucleus of the solitary tract (NTS), and the dorsal motor nucleus of the vagus (DMV) (Fig. 3C). IRS4^PVH^ neurons also send projections to the median eminence, a site important for endocrine control of pituitary function (Fig. 3D). In addition to hindbrain regions, syn-mCherry projections were identified in the intermediolateral column (IML) of the thoracic spinal cord in close proximity to choline acetyltransferase (ChAT)-producing neurons, suggesting a circuit mechanism for the regulation of sympathetic activity by IRS4^PVH^ neurons (Fig. 3E). Of note, these results are strikingly similar to projections originating from NOS1^PVH^ neurons, despite the fact that these populations do not appear to overlap in the PVH^10^.

### Projection-specific modified rabies tracing identifies unique inputs to IRS4^PVH^ subpopulations

**Figure 4:**
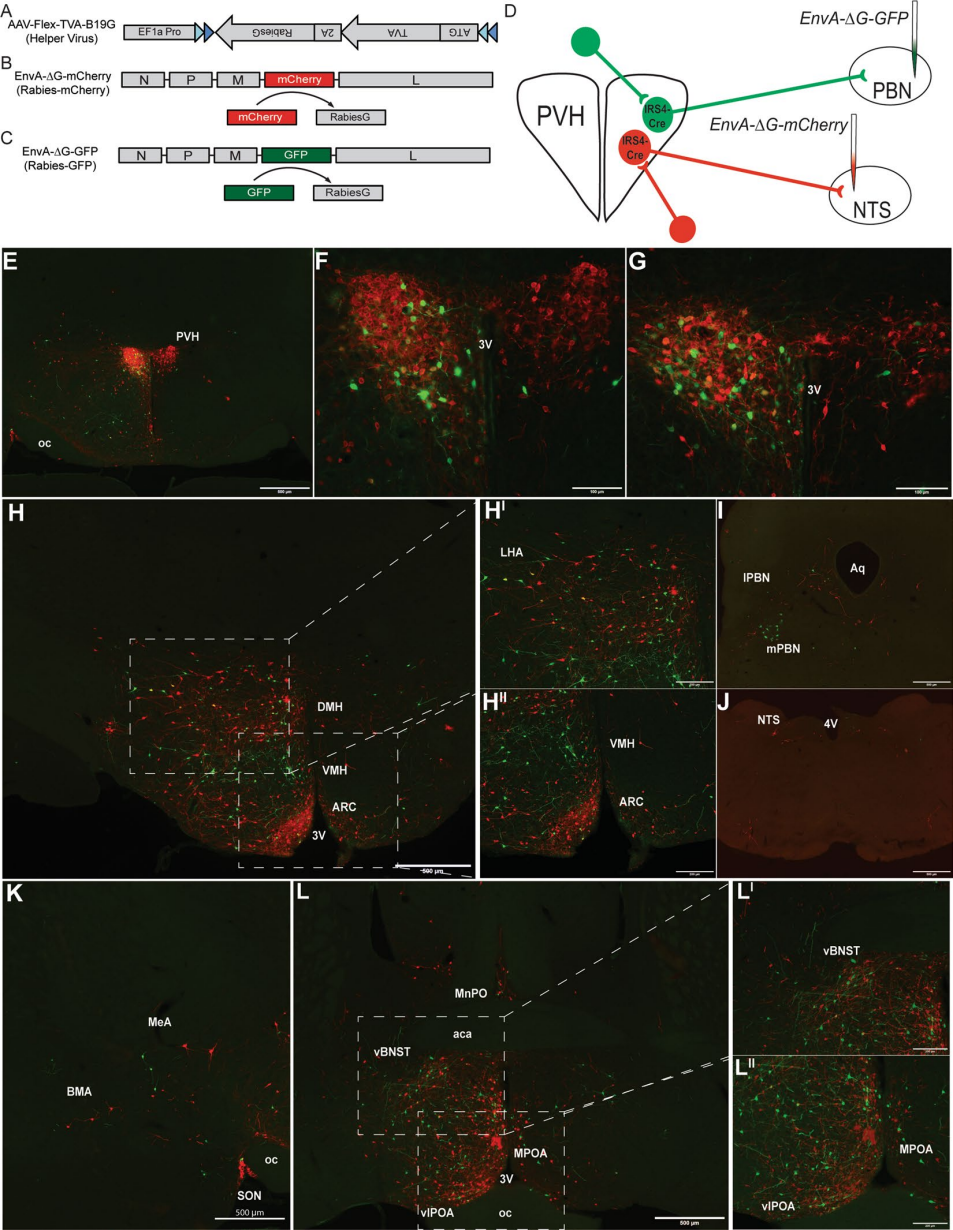
Identification of monosynaptic inputs to NTS-projecting or PBN-projecting IRS4^PVH^ neurons using modified rabies virus. A Cre-dependent helper virus construct (AAV-Flex-TVA-B19G) is used to insert rabies B19 glycoprotein (B19G) and the TVA receptor in IRS4^PVH^ cell bodies and terminals. Modified rabies virus expresses a fluorescent tag (mCherry, **B**; GFP, **C**) instead of B19G. After initial infection with helper virus, rabies-mCherry is injected at one projection site (NTS), whereas rabies-GFP is injected at another (PBN) in the same mouse (**D**). IHC for mCherry and GFP identify largely non-overlapping NTS-projecting and PBN-projecting IRS4^PVH^ neurons, respectively (**E**–**G**). Sites upstream of both NTS and PBN-projecting IRS4^PVH^ neurons include the lateral hypothalamic area (LHA, **H**), supraoptic nucleus (SON, **K**), amygdala (**K**), bed nucleus of the stria terminalis (BNST, L), and the preoptic area (POA, L). The ventromedial hypothalamus (VMH) is upstream of PBN-projecting IRS4^PVH^ neurons (H^II^, green), whereas both the arcuate nucleus (ARC, H^II^, red) and PBN (I, red) are upstream of IRS4^PVH^ neurons projecting to the NTS. Glial damage represents injection site in the PBN (I, green) and NTS (J, red). 3 V = third ventricle, oc = optic chiasm, MeA = medial amygdala, BMA = basomedial amygdala, vBNST = ventral BNST, vlPOA = ventral lateral POA, MPOA = medial POA, MnPO = median preoptic nucleus, aca = anterior part of anterior commissure, DMH = dorsomedial hypothalamus, aq = aqueduct, 4 V = fourth ventricle.

As IRS4^PVH^ neurons project to various brain regions implicated in feeding and energy expenditure, we hypothesized that the afferent input to distinct IRS4^PVH^ circuits might differ based on IRS4^PVH^ neuronal projection site. To test this hypothesis, we labeled monosynaptic inputs to either NTS-projecting or PBN-projecting IRS4^PVH^ neurons in the same animal using a modified rabies virus approach that requires Cre-dependent helper virus (AAV-Flex-TVA-B19G) expression. Due to the limited efficacy and applicability of available helper virus reagents for our desired approach, we generated a Cre-dependent helper virus that co-expresses both the TVA receptor and B19 glycoprotein (B19G) via a self-cleaving 2A peptide linker (Fig. 4A). Modified rabies virus (EnvA-ΔG-mCherry (rabies-mCherry, Fig. 4B) or EnvA-ΔG-GFP (rabies-GFP, Fig. 4C)) cannot enter cells without the TVA receptor and is modified to express a fluorescent protein (mCherry or GFP) instead of B19G^16,17^. Therefore, infection of the modified rabies virus requires viral expression of the TVA receptor and retrograde transport of rabies-mCherry or rabies-GFP requires viral expression of the B19G (Fig. 4D). The fidelity of this system is demonstrated by a lack of rabies-mCherry expression in the brain of *Sim1-Cre* mice in which the helper virus injection missed the PVH (determined by a lack of 2A staining in the PVH), despite rabies-mCherry injection into the NTS (Supp. Fig. 4.1A,B). In this approach, neurons upstream of primary infected neurons theoretically do not express Cre recombinase, and therefore do not produce B19G; this limits rabies-mCherry or rabies-GFP expression to monosynaptic inputs to IRS4^PVH^ neurons.

Cre-dependent expression of the TVA receptor throughout the cell body and synaptic terminals of transduced neurons allows terminal-specific rabies infection^18^. To determine if subpopulations of IRS4^PVH^ neurons might be engaged by discrete afferent inputs, we performed dual rabies virus injections at anatomically separable projection targets in the same mouse (Fig. 4D). Three weeks following helper virus injection, rabies-mCherry was injected in the NTS whereas rabies-GFP was injected in the PBN. Following 5 days of incubation, we found that IRS4^PVH^ neurons that project to the NTS or PBN are largely distinct as evidenced by the lack of significant fluorescence colocalization in the PVH (colocalized cells: n = 27 ± 5.7 cells, n = 2 mice). The fact that some co-localization between GFP and mCherry occurs in the PVH suggests the existence of PVH neurons capable of regulating both the NTS and PBN presumably either through collateralization or intra-PVH microcircuitry (Fig. 4E–G).

Consistent with published literature examining PVH circuitry, our rabies monosynaptic labeling demonstrates that the arcuate nucleus (ARC) is an upstream site of NTS-projecting IRS4^PVH^ neurons (Fig. 4H,H^II^)^19^. Additional hypothalamic sites engaging NTS-projecting IRS4^PVH^ neurons include the lateral hypothalamic area (LHA, Fig. 4H^I^) and the dorsomedial hypothalamus (DMH, Fig. 4H), whereas sites upstream of PBN-projecting IRS4^PVH^ neurons largely include the ventromedial hypothalamus (VMH) and LHA (Fig. 4H). Although monosynaptic rabies tracing is not a quantitative methodology, a small number of LHA neurons co-express GFP and mCherry, suggesting LHA modulation of both IRS4^PVH^-PBN and IRS4^PVH^-NTS circuits (Fig. 4H^I^, n = 8 ± 11.3 cells, n = 2 mice). In contrast, very few afferent neurons in the ARC co-express GFP and mCherry (n = 2 ± 2.1 cells, n = 2 mice). Forebrain sites including the preoptic area (POA, Fig. 4L^II^) and bed nucleus of the stria terminalis (BNST, Fig. 4L^I^) include largely non-overlapping populations upstream of both IRS4^PVH^ circuits, suggesting distinct forebrain circuits may modulate IRS4^PVH^ neuronal function.

### IRS4^PVH^ neurons are necessary for normal feeding and energy balance

**Figure 5:**
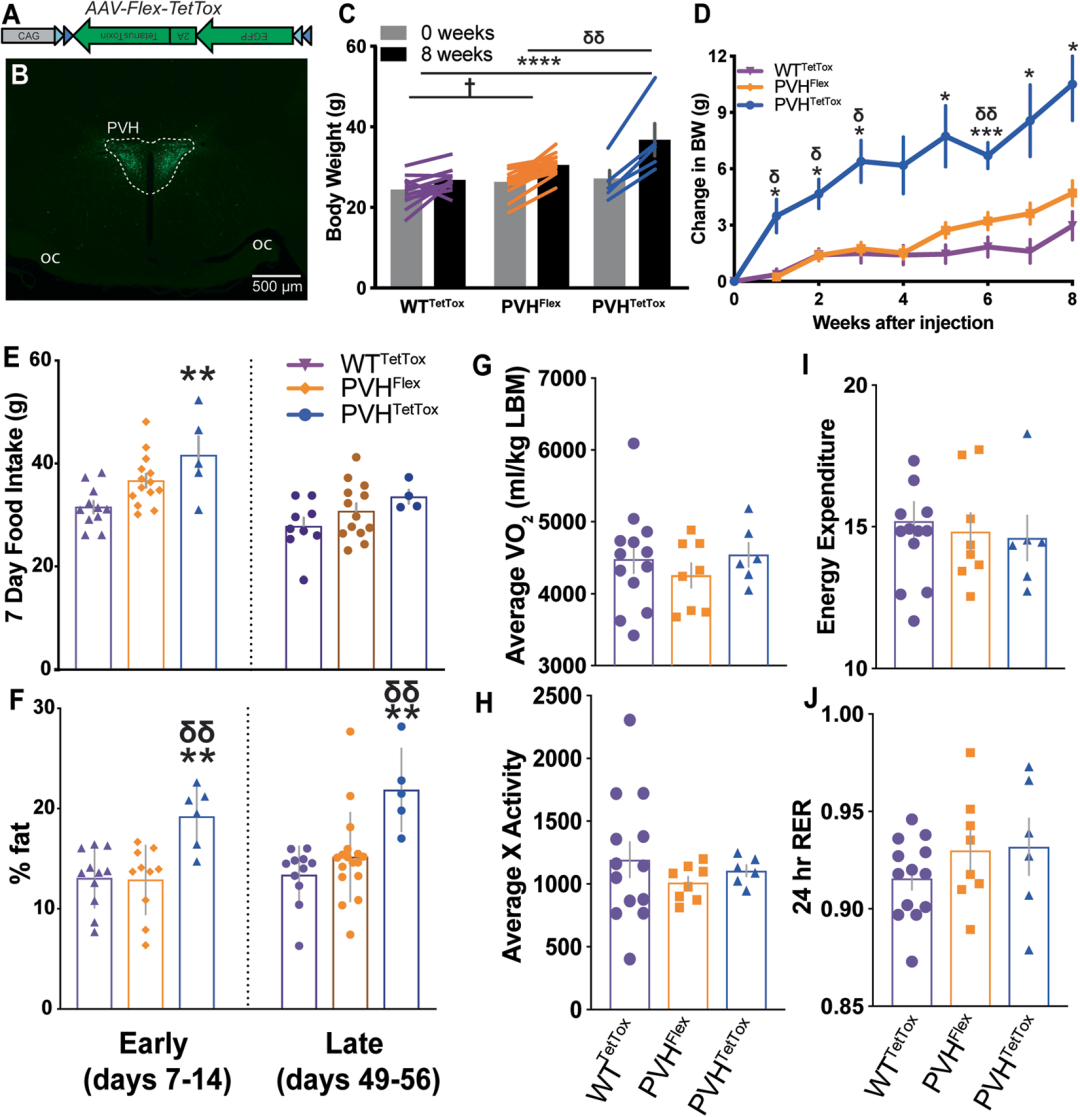
IRS4^PVH^ neurons are necessary for normal feeding and bodyweight. A Cre-dependent adeno-associated virus expresses tetanus toxin A and GFP exclusively in Cre-expressing neurons to inhibit synaptic vesicle exocytosis and GFP visualization of the transduced cells (**A**). (**B**) Example hit site of *IRS4-iCre* mice injected with AAV-Flex-TetTox (PVH^TetTox^). Bilateral IRS4^PVH^ neuronal silencing in *IRS4-iCre* mice (PVH^TetTox^) results in both increased bodyweight (**C**) and increased bodyweight gain (**D**) in comparison to *IRS4-iCre* mice injected with a control AAV (PVH^Flex^) or wildtype mice injected with AAV-Flex-TetTox (WT^TetTox^, mixed-model analysis followed by Tukey’s post-hoc). (**E**) PVH^TetTox^ mice (blue) show increased 7-day food intake at early stages of the study (left) in comparison to WT^TetTox^ mice (orange; one-way ANOVA followed by Tukey’s post-hoc) whereas later stage food intake shows a trend towards increased feeding (dark blue, one-way ANOVA). (**F**) PVH^TetTox^ mice show have increased fat mass percentages (**F**) at early and late stages of obesity development (one-way ANOVA followed by Tukey’s post-hoc). (**G**–**J**) 3-day averages of CLAMS measurements were measured during weeks 2–4 of experiments and compared across groups. While PVH^TetTox^ mice show a trend towards decreased energy expenditure (**I**), none of these groups are significantly different from one another (one-way ANOVA. Average values ± SEM are shown, *p < 0.05, **p < 0.01, ***p < 0.001, ****p < 0.0001 compared to WT^TetTox^; ^Ƹ^p < 0.05, ^Ƹ Ƹ^p < 0.01, ^Ƹ Ƹ Ƹ^p < 0.001 compared to PVH^Flex^; BW/feeding: PVH^TetTox^ n = 6, PVH^Flex^ n = 17, WT^TetTox^ n = 13; body composition: PVH^TetTox^ early n = 6, PVH^TetTox^ late n = 5, PVH^Flex^ early n = 10, PVH^Flex^ late n = 17, WT^TetTox^ n = 11; CLAMS: PVH^TetTox^ n = 6, PVH^Flex^ n = 8, WT^TetTox^ n = 13.

To test the necessity of IRS4^PVH^ neuron activity in physiologic body weight regulation, we permanently silenced IRS4^PVH^ neurons using a Cre-dependent tetanus toxin virus (AAV-Flex-TetTox) that cleaves the SNARE protein, synaptobrevin, and inhibits synaptic vesicle release^20^. This construct has been modified to express the A subunit and does not travel retrogradely, therefore limiting neuronal silencing to IRS4 Cre-expressing neurons^21^. We performed bilateral PVH injections of AAV-Flex-TetTox in *IRS4-iCre* mice (Fig. 5A,B). In comparison to control Cre-dependent viral injections into the PVH of *IRS4-iCre* mice (PVH^Flex^) or AAV-Flex-TetTox injections in the PVH of wildtype mice (WT^TetTox^), mice with IRS4^PVH^ neuronal silencing show modest obesity within one week of injection (Fig. 5C: PVH^TetTox^ v. WT^TetTox^ p = 0.0162; Fig. 5D: PVH^TetTox^ v. WT^TetTox^ p = 0.0008, PVH^TetTox^ v. PVH^Flex^ p = 0.0003; mixed effects analysis followed by Tukey’s post-hoc). Obesity following elimination of IRS4^PVH^ signaling is likely driven primarily by early hyperphagia, demonstrated by increased food intake in the second week of the study (Fig. 5E, PVH^TetTox^ v. WT^TetTox^ p = 0.0303; one-way ANOVA followed by Tukey’s post-hoc). Mice with silenced IRS4^PVH^ neurons demonstrate a non-significant trend towards decreased energy expenditure during the second and third weeks of the study (Fig. 5G,I,J, energy expenditure p = 0.074; RER p = 0.075; one-way ANOVA), without changes in total X activity (Fig. 5H). Body composition analysis indicates that IRS4^PVH^ neuronal silencing leads to an increased fat percentage at both early (Fig. 5F, left; PVH^TetTox^ v. PVH^Flex^ p = 0.0259, one-way ANOVA followed by Tukey’s post-hoc) and late (Fig. 5F, right; PVH^TetTox^ v. WT^TetTox^ p = 0.0377; one-way ANOVA followed by Tukey’s post-hoc) time points after injection. Unilateral IRS4^PVH^ neuronal silencing was also associated with increased bodyweight throughout the course of the experiment, with trends toward similar effects on feeding and energy expenditure (Supp. Table 5.1, one-way ANOVA followed by Tukey’s post-hoc if applicable).

### IRS4^PVH^ neurons are dispensable for melanocortin-induced feeding suppression

**Figure 6:**
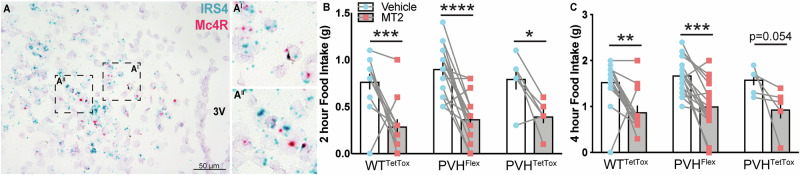
IRS4^PVH^ neuron activity is dispensable for melanocortin agonist-mediated satiety. *In situ* hybridization for *Irs4* (**A**, blue) and *Mc4r* (**A**, red) mRNA in the PVH of adult wildtype mice. Melanocortin agonist-induced satiety was tested in mice with IRS4^PVH^ neuronal silencing (**B**,**C**). Two-hour (**B**) and four-hour (**C**) feeding was significantly decreased following i.p. injection of the MC4R agonist MT-II (150 ug/mouse) in all cohorts of mice, despite the silencing of IRS4^PVH^ neurons in the PVH^TetTox^ group. Average values ± SEM are shown, **p < 0.01, ***p < 0.001, ****p < 0.0001; PVH^TetTox^ n = 6, PVH^Flex^ n = 17, WT^TetTox^ n = 13. Significance was determined using a paired t-test between vehicle and MTII injected mice within the same cohort. Dashed boxes (A^I^, A^II^) indicate regions cropped and digitally enlarged.

Given the important role of the PVH in mediating melanocortin-induced satiety, and the identification of numerous ARC inputs to IRS4^PVH^ neurons, we hypothesized that IRS4^PVH^ neurons might contain melanocortin 4 receptors (MC4R) and participate in melanocortin agonist-induced anorexia. To test this, we performed *in situ* hybridization for *Mc4r* and *Irs4* in wildtype mice (Fig. 6A). Although most IRS4^PVH^ neurons do not contain *Mc4r* mRNA (9.67% + 1.11% of IRS4^PVH^ with *Mc4R*, n = 3 mice), a significant fraction of MC4R^PVH^ neurons do express *Irs4* mRNA (53.66% + 1.13%). To test the physiologic importance of melanocortin action in IRS4 neurons, we attempted to delete MC4R expression from IRS4 neurons by crossing *IRS4-iCre* and the Cre-dependent *lox-Mc4R* mouse line^7^. Unfortunately, developmental expression of Cre recombinase from the *IRS4-iCre* allele resulted in germline deletion of *Mc4R* (data not shown). We then tested the ability of the melanocortin agonist melanotan-II (MTII) to suppress dark cycle feeding in mice with AAV-Flex-TetTox or control injections in the PVH at 4–5 weeks post-injection. Despite the inability to transmit information from IRS4^PVH^ neurons (and therefore a significant portion of MC4R^PVH^ neurons), MTII injection (150 ug/mouse) still suppressed both two and four hour feeding in all cohorts of mice, suggesting that IRS4^PVH^ neuron activity is not required for the melanocortin feeding response (Fig. 6B,C; two-way repeated measures ANOVA vehicle vs. MT2; two hour PVH^TetTox^ p = 0.0312, t = 2.714, WT^TetTox^ p = 0.0001, t = 4.763, PVH^Flex^ p < 0.0001, t = 6.113; four-hour PVH^TetTox^ p = 0.0535, t = 2.486, WT^TetTox^ p = 0.0025, t = 3.681, PVH^Flex^ p = −0.0004, t = 4.355).

## Discussion

The combination of genetic mouse models with site-specific delivery of chemogenetic and optogenetic tools has greatly advanced our understanding of the role of the PVH in modulating energy balance through feeding and/or energy expenditure. Nonetheless, few studies have genetically identified specific PVH populations crucial for these functions. Our previous work suggests the importance of a non-OXT Nos1^PVH^ population in feeding regulation^10^. Here, we identify a different (non-OXT and non-NOS1^PVH^) population expressing insulin receptor substrate-4 *(Irs4)* that is necessary for normal bodyweight maintenance.

Genetic deletion of *Irs4* results in modest obesity and altered glucose homeostasis; whether this phenotype reflects simply a loss of *Irs4* function or developmental compensation in response to *Irs4* loss is unknown^12,22^. In our approach, we used site and temporal specific manipulations in adult organisms to circumvent developmental issues and directly examine the function of IRS4-expressing cells predominantly within the PVH. Whereas chemogenetic activation reveals the potential function of IRS4^PVH^ neuronal function in adult mice, neuronal silencing with tetanus toxin addresses the physiologic necessity of this population in various energy balance parameters. Indeed, permanent inhibition of IRS4^PVH^-specific synaptic release results in obesity due to altered feeding and a trend toward lower energy expenditure.

An important caveat to this study flows from its methodologic approach. Stereotaxic injections to defined brain regions are not always exclusively limited to cells within a classically defined neuroanatomical region. In our study we have excluded data for physiologic analyses from animals with significant extra-PVH viral transduction. All analyses were performed using data from animals with limited extra-PVH viral expression. However, post-hoc immunocytochemistry of brain sections from analyzed animals revealed transduction of IRS4 Cre-expressing cells adjacent to the PVH. The contribution of these subparaventricular and periventricular IRS4 cells to the outputs measured cannot be determined from the current study. In our study there were no cases of PVH-targeted injections that were limited to just the subparaventricular or periventricular IRS4-expressing cells that would allow physiologic comparisons with the PVH and peri-PVH-transduced cohort. Highly refined injections limited to cells solely in the PVH or solely in adjacent areas would be needed to completely define the contributions of these peri-PVH IRS4-expressing cells to energy balance control.

Cre-dependent efferent tract tracing suggests that the circuitries engaged by IRS4^PVH^ neurons used to regulate feeding and energy expenditure are similar to those identified for both NOS1^PVH^ and MC4R^PVH^ neurons and include the PBN and NTS and sparse projections to the spinal cord^7,10,11^. While our current studies cannot discriminate which projection site is relevant for controlling feeding and energy expenditure, it is possible that IRS4^PVH^ neurons projecting to the PBN contribute to feeding modulation since the PBN is the relevant output in MC4R-mediated feeding suppression^11^. However, our data indicate that IRS4^PVH^ neurons and MC4R^PVH^ neurons overlap only in part, and that MC4R-mediated feeding suppression is unaffected by IRS4^PVH^ neuronal silencing. Therefore, it remains possible that other projection output sites may drive the IRS4^PVH^-mediated satiety response.

Given their minimal overlap in the PVH, the functional redundancy of the IRS4^PVH^ and NOS1^PVH^ neuronal populations in energy expenditure and feeding control is intriguing. A recent report suggests that nonoverlapping MC4R^PVH^ and PDYN^PVH^ neuron populations function together to coordinate a full PVH satiety response^23^. The contribution of other non-MC4R PVH neurons in energy expenditure control is less clear. Here, we demonstrate that despite neuronal silencing of IRS4^PVH^ neurons, which includes half of the MC4R^PVH^ population, the satiety response to the melanocortin agonist MT-II remains intact. Thus, the anorectic response to melanocortin agonist is not dependent on IRS4^PVH^ neurons, even though they compromise ~50% of the MC4R^PVH^ population. Although most IRS4^PVH^ neurons do not express *Pdyn*, chemogenetic activation of IRS4^PVH^ will activate a subset of PDYN^PVH^ neurons. Whether this subset is responsible for the feeding effects seen following IRS4^PVH^ activation remains to be determined. IRS4 and TRH overlap significantly in the PVH. However, feeding suppression following IRS4^PVH^ activation is unlikely mediated by TRH^PVH^ neurons since activation of this population has been shown to promote feeding by targeting orexigenic AgRP neurons in the ARC^24^. Future studies will be needed to determine whether the IRS4/TRH^PVH^ subset modulates energy expenditure through direct effects on autonomic output or alterations in the pituitary-thyroid axis. In addition, whether distinct PVH populations control feeding and energy expenditure via disparate projection sites (e.g. PBN vs. NTS vs. spinal cord) requires additional investigation.

The PVH is a hypothalamic relay station for energy balance control. It receives dense innervation from sites critical for feeding and energy expenditure regulation, integrates this incoming energy balance information and then transmits an output signal to hindbrain sites to achieve appropriate physiologic and behavioral responses^19,25–27^. Whether PVH inputs can be mapped on to distinct PVH outputs (either anatomical or physiological) is not known. Here, we used monosynaptic rabies tracing to identify the possibility of unique afferent inputs to NTS-projecting versus PBN-projecting IRS4^PVH^ neurons. We demonstrate that NTS-projecting IRS4^PVH^ neurons receive dense innervation from the ARC, confirming this approach in a historically defined circuit. Additionally, PBN-projecting IRS4^PVH^ neurons receive innervation from the VMH. Given the role for direct PVH connections to the PBN in mediating MC4R-induced satiety, it is possible that the VMH-IRS4^PVH^-PBN circuit is relevant in feeding control, though future studies will be necessary to test the function of this circuit and better characterize its genetic signature^11^. It is of interest that a few LHA neurons appear to be upstream of both PBN and NTS-projecting IRS4^PVH^ neurons. Given the role for the LHA in controlling both energy expenditure and feeding behavior, this raises the possibility that the PVH input neurons originating in the LHA might be coordinating distinct energy balance parameters via divergent IRS4^PVH^ circuits^28–30^.

PVH neurons are predominantly glutamatergic and highly interconnected through PVH interneurons that may coordinate neural activity between PVH subsets^31–33^. Our modified rabies tracing findings suggest that projection-defined IRS4^PVH^ neurons are likely innervated by other local PVH populations based on the dense expression of rabies virus throughout the PVH (Fig. 4E–G). Since some PVH populations suppress feeding without affecting energy expenditure (i.e. MC4R^PVH^), whereas others increase energy expenditure absent of feeding regulation (i.e. OXT^PVH^), it seems likely that separate circuitries exist to coordinate these nodes. By extension, this suggests that some PVH populations (such as OXT^PVH^) are differentially connected and/or insulated from other PVH circuits in terms of feeding regulation. One potential model for PVH action could include a final common output from the PVH that is responsible for coordinating feeding regulation. In such a scenario, NOS1^PVH^ and/or IRS4^PVH^ neurons might lie upstream of or parallel to MC4R^PVH^ neurons and coordinate feeding suppression via projections to the PBN. Certainly, connections between separate PVH populations are well documented^34–36^. Moreover, the dense PVH interconnectivity highlighted by projection-specific monosynaptic retrograde tracing suggests the possibility that anatomically and functionally separable IRS4^PVH^ neuronal circuits can be coordinately regulated in order to promote an orchestrated physiologic output through simultaneous transmission to multiple brain sites.

Taken together, our results clearly demonstrate that IRS4^PVH^ neurons are a genetically defined PVH population capable of controlling feeding behavior and overall energy balance, presumably through projections to hindbrain and spinal cord regions. Moreover, tetanus toxin sensitive vesicle fusion in IRS4^PVH^ cells is essential in preventing hyperphagia and obesity. Furthermore, our studies support the concept of a complex, intra-PVH network that regulates hindbrain structures previously shown to control energy balance parameters. While the significance of this local communication between PVH subpopulations in the control of distinct aspects of energy balance remains to be elucidated, further characterization of the composition and connectivity of individual PVH populations is necessary to fully understand the control of feeding and energy expenditure by an essential hypothalamic output center.

## Materials and Methods

### Experimental animals

*IRS4-ires-Cre* (*IRS4-iCre*) mice were generated using methods previously described^37^. Briefly, genomic DNA including the 3’ UTR of the murine *Irs4* gene was PCR amplified from R1 ES cells and cloned into a plasmid for insertion of an Frt-flanked neomycin selection cassette followed by an internal ribosomal entry sequence fused to a Cre recombinase transgene (iCre) between the STOP codon and the polyadenylation site. Constructs were linearized and electroporated into R1 ES cells by the University of Michigan Transgenic Animal Model Core. Correctly targeted ES cells were identified by quantitative real-time PCR and Southern blots and then injected into C57Bl/6J blastocysts to generate chimeric animals. Chimeras were then bred to C57Bl/6J females to confirm germline transmission and generate the *IRS4-neo-iCre* mice. To remove the Frt-flanked neo cassette, IRS4-neo-iCre mice were then bred to Flp deleter mice (Jax 012930).

Adult male mice (8–16 weeks old) were used for all studies. *Irs4* is located on the X-chromosome which confounds experiments using *IRS4-iCre* in females given the random lyonization of the X chromosome. All animals were bred and housed within our colony according to guidelines approved by the University of Michigan Committee on the Care and Use of Animals. Unless otherwise noted, mice were provided *ad libitum* access to food and water. All mice were acclimatized to intraperitoneal (i.p.) injections three days prior to any experimental i.p. injection.

### Generation of modified rabies virus

Replication deficient modified rabies virus containing fluorescent reporters in place of the B19 glycoprotein (EnvA-ΔG-mCherry and EnvA-ΔG-GFP) were generated in the University of Michigan viral vector core using conditions previously described^16,17^.

### Stereotaxic injections

Stereotaxic injections were performed in *IRS4-iCre* and non-transgenic (WT) mice as previously described^10^. Briefly, mice were placed in a digital stereotaxic frame (Model 1900, Kopf Instruments) under isofluorane and provided with pre-surgical analgesia. Viral injections were performed using a pressurized picospritzer system coupled to a pulled glass micropipette (coordinates from bregma: PVH: A/P = −0.500, M/L = +/− 0.220, D/V = −4.800). For tract tracing experiments, 50 nl of the adenoviral synatophysin-mCherry terminal tracer (Ad-iN/syn-mCherry^38^) was unilaterally injected in *IRS4-iCre* mice. For functional analysis of IRS4^PVH^ neurons, bilateral PVH injections of AAV-Flex-hM3Dq-mCherry (AAV-Flex-hM3Dq, purchased from UNC Vector Core), AAV-Flex-TetTox (purchased from the Stanford Viral Vector core) or control injections of AAV-Flex-GFP were performed in *IRS4-iCre* or WT mice (50 nl/side). For analysis of monosynaptic upstream inputs to IRS4^PVH^ neurons, *IRS4-iCre* mice were unilaterally injected with AAV-Flex-TVA-B19G in the PVH and allowed to recover for at least 21 days to ensure adequate helper virus expression throughout both cell bodies and terminals^39^. Mice then underwent a second surgery with dual stereotaxic injections into ipsilateral PVH projection targets with rabies-GFP in the PBN (A/P = −4.770, M/L = +/− 1.35, D/V = −2.8) and rabies-mCherry injected into the NTS. NTS injections were performed as previously described, whereby the fourth ventricle was identified and used as a geographic landmark to determine the site of injection^10^. A glass micropipette was lowered into the site (D/V: −0.550) and ~25 nl of virus was injected. Mice injected with the Ad-iN/syn-mCherry tracer were individually housed for five days following injection to allow for viral transduction and protein transport before perfusion, whereas mice injected with modified rabies virus were perfused seven days following rabies virus injections. Mice injected with AAV-Flex-hM3Dq were allowed to recover for fourteen days following surgery before further experiments were performed.

### Effect of PVH^IRS4^ neuronal activation on feeding and energy expenditure

Following recovery, *IRS4-iCre* + AAV-Flex-hM3Dq mice underwent feeding and energy expenditure assays as previously described^10^. Briefly, to measure changes in energy expenditure, *IRS4-iCre* + AAV-Flex-hM3Dq mice were acclimatized for two consecutive days to the Comprehensive Laboratory Monitoring System (CLAMS, Columbus Instruments) in the University of Michigan’s Small Animal Phenotyping Core to obtain multi-parameter analysis including open circuit calorimetry and activity via optical beam breaks. Following acclimatization, food was removed from metabolic cages during the light cycle on experimental days beginning two hours prior to experiments. Mice received an i.p. injection of vehicle (10% β-cyclodextrin, Sigma) and CLAMS measurements were analyzed for the following four hours. Mice remained in the chambers with food access at the onset of the dark cycle and the experiment repeated at the same time the following day instead with i.p. injection of CNO (0.3 mg/kg in 10% B-cyclodextrin). While measurements were performed throughout the duration of the experiment, data shown are averaged over the 4 hours following injection of vehicle or CNO. Resting energy expenditure data shown was determined by analyzing data oxygen consumption values at data points in which activity levels were approximately matched during the four hours before and after CNO administration. In experiments aimed to identify feeding changes induced by IRS4^PVH^ neuronal activation, mice were fasted during the day and received an i.p. injection of vehicle at the onset of the dark cycle with the presentation of food. Food intake was measured at two and four hours after injection and the experiment repeated the following day upon injection of CNO at the onset of the dark cycle. In a separate cohort of mice, the effects of CNO were tested on wildtype mice injected with AAV-Flex-hM3Dq and compared to *IRS4-iCre* mice injected with AAV-Flex-hM3Dq. In these experiments, CNO or vehicle (0.9% sodium chloride) injections were counterbalanced and performed 4 days apart.

### Longitudinal bodyweight, food intake, and calorimetry measurements

Mice injected with AAV-Flex-TetTox or control viruses were allowed to recover for 7 days before weekly body weight and food intake measurements began. Energy expenditure was determined using the Comprehensive Laboratory Monitoring System (CLAMS, Columbus Instruments) in the University of Michigan’s Small Animal Phenotyping Core to obtain multi-parameter analysis including open circuit calorimetry and activity via optical beam breaks between 11–24 days post-injection. Mice were allowed to acclimatize to the chambers for two days, followed by VO_2_ and locomotor activity data collection for three consecutive days. Analysis of CLAMS data was averaged across all three days of recording. Body composition analysis (Minispec LF90 II, Bruker Optics) was performed at both two weeks and 7 weeks following injection.

### MT-II-induced feeding suppression

Five weeks following viral injection, *IRS4-iCre* mice injected with AAV-Flex-TetTox and appropriate controls were fasted during the light cycle (10:00–18:00). At the onset of the dark cycle, mice received an i.p. injection of 0.9% sodium chloride (APP Pharmaceuticals, 63323-186-10) or melanotan-II (MT-II, 150 ug/mouse, Bachem) and *ad libitum* access to food. Food intake was measured two and four hours post injection. The following week, injections were counter balanced and corresponding food intake measured.

### Perfusion and Immunohistochemistry (IHC)

At the end of all experiments, mice were perfused to verify viral injection sites. Briefly, mice were deeply anesthetized with an overdose of pentobarbital (150 mg/kg, IP) and transcardially perfused with sterile PBS followed by 10% neutral buffered formalin or 4% paraformaldehyde (for perfusions with spinal cord removal. Brains and spinal cords (syn-mCherry injections only) were removed, post-fixed, and dehydrated in 30% sucrose before sectioning into 30 μm slices on a freezing microtome (Leica). Coronal brain sections were collected in four representative sections whereas longitudinal thoracic spinal cord sections were collected in three representative sections and stored at −20 °C. For 2A peptide and cFos immunohistochemistry (IHC), free floating brain and spinal cord sections were pretreated with 30% H_2_O_2_ to remove endogenous peroxidase activity and then blocked with normal goat or donkey serum and incubated in primary antibody overnight (rabbit anti-2A 1:1,000, Millipore ABE457; rabbit anti-cFos). Detection of primary antibody was performed by avidin-biotin/diaminobenzidine (DAB) method (Biotin-SP-conjugated Donkey Anti-Rabbit, Jackson Immunoresearch, 1:200; ABC kit, Vector Labs; DAB reagents, Sigma). mCherry and choline acetyltransferase (ChAT) were detected using with primary antibodies for dsRed (rabbit 1:1000, Clontech, 632496) or TdTomato (rat, 1:1000, Kerafast 16D7) and ChAT (spinal cords only; goat, 1:500, Millipore AB144P) respectively followed by secondary immunofluorescence detection with donkey anti-rabbit-Alexa 568 or donkey anti-goat-Alexa 488 (1:200, Invitrogen). For PVH colocalization, rabies experiments, cFos (non-DAB method) and TetTox-GFP expression, IHC immunostaining was performed using primary antibodies for cFos (rabbit, 1:1000, Cell Signaling 9F6), GFP (rabbit 1:20,000, Invitrogen A6455), nNos1 (sheep 1:1500, (24), kindly provided by Dr. Vincent Prevot), neurophysin (goat, 1:1000, Santa Cruz Biotechnology, sc-7810) and copeptin (goat 1:1000, Santa Cruz Biotechnology, sc-7812). For all AAV-TetTox and AAV-hM3Dq injections, bilateral or unilateral PVH hit sites were verified and misses eliminated from data analysis, with viral injections that modestly leaked into the peri-PVH included in the PVH groups. Viral leak into any other area outside of this region were excluded. Imaging was performed using an Olympus BX-53 upright microscope with G6000 camera.

### *In situ* hybridization

Mice were deeply anesthetized with an overdose of inhaled isofluorane and brains removed and flash frozen in 2-methylbutane. Brains were sectioned into 16 μm sections onto glass slides using a cryostat (Leica CM 1950) and stored at −80 °C. The probes were purchased from Advanced Cell Diagnostics, and the assays were performed according to the manufacturer’s protocol. The sections were fixed in cold 10% formalin for 1 hour, followed by dehydration in 50% and 75% ethanol for 5 minutes each, and 100% ethanol twice. Sections were dried at 40 °C for 30 minutes. For fluorescent staining, sections were pretreated in protease IV for 30 minutes, washed in PBS twice, and then incubated with the desired probes for 2 hours and then washed twice in 1x wash buffer (ACD, 310091) for 2 minutes each. Amplification and detection steps were performed using the RNAscope® Fluorescent Multiplex Reagent Kit (320850). Sections were incubated with Amp1 for 30 min, Amp2 for 15 min, Amp3 for 30 min, and Amp 4 Alt C for 15 min. There were 2 washes between each amplification, and all amplifications were performed at 40 °C in the EZ Hybridization oven. To demonstrate overlapping expression of IRS4 and Cre mRNA, IRS4 and Cre probes were used. To quantify overlapping expression of IRS4 and CRH, PDYN and TRH, fluorescent probes were used. For the tissues exposed to the IRS4, CRH, PDYN, TRH and Cre probes, the slides were stained with DAPI for 30 seconds before coverslipping using Prolong Gold antifade reagent (Invitrogen). To investigate MC4R and IRS4 expression in the PVH, chromogen staining was conducted using the RNAscope® 2.5 HD Duplex Reagent Kit (ACD 320701). The sections were treated with H_2_O_2_ for 10 min and then incubated with the protease K IV for 30 min. After hybridization with probes against MC4R and IRS4 mRNA, the sections were washed 2 times, followed with amplification from Amp1 to Amp6 with 2 washes in between. To detect the red signal component, Red-B was diluted 1:60 in component Red-A and incubated on the tissue for 10 minutes at room temperature. Slides were then rinsed two times in wash buffer to stop the chromogen reaction. Amplification continued with Amp7 through Amp10, followed by detection of the green signal, which was achieved by diluting component Green-B 1:50 in component Green-A and incubating for 10 minutes at room temperature. Counterstaining was performed by immersing the slides in 50% hematoxylin for 30 seconds. The slides were then dried and mounted in VectaMount mounting medium (Vector Laboratories, INC). Imaging for fluorescent staining was performed using an Olympus BX-53 microscope with a G6000 camera. Imaging for the chromogen staining used an Olympus BX-51 microscope with a DP80 camera (Olympus). For the purposes of quantification, images from the coronal sections were processed uniformly using Photoshop (Adobe) to remove background and to mark cells that expressed a given probe. Cells (indicated by DAPI/hematoxylin stain) determined to be positive for each probe were quantified using ImageJ and summed for each mouse, with the percentages of overlapping expression averaged between the mice.

### Statistical analysis

Paired t-tests, one-way ANOVAs followed by Tukey post-hoc tests (if applicable), two-way ANOVAs followed by Sidak’s multiple comparisons (if applicable), or mixed model analyses were calculated using GraphPad Prism 8 as appropriate. Significance was determined for p < 0.05.

## Supplementary information

Supplementary Information.

## Data Availability

All data generated or analysed during this study are included in this published article (and its Supplementary Information files).
