# Supplementary material for: Paraventricular, subparaventricular and periventricular hypothalamic IRS4-expressing neurons are required for normal energy balance
Source: Sci Rep. 2020 Mar 26;10:5546. doi: 10.1038/s41598-020-62468-z (PMC7099088; doi:10.1038/s41598-020-62468-z)
Supplement: Supplementary file 1 — Supplementary Information. [file 41598_2020_62468_MOESM1_ESM.pdf]

Supplementary Information for

"Paraventricular, subparaventricular and periventricular hypothalamic IRS4-expressing neurons are required for normal energy balance"

Amy K. Sutton*, Ian E. Gonzalez*, Marianna Sadagurski, Michael Rajala, Chunxia Lu, Margaret B. Allison, Adams JM, Martin G. Myers, Morris F. White, David P. Olson

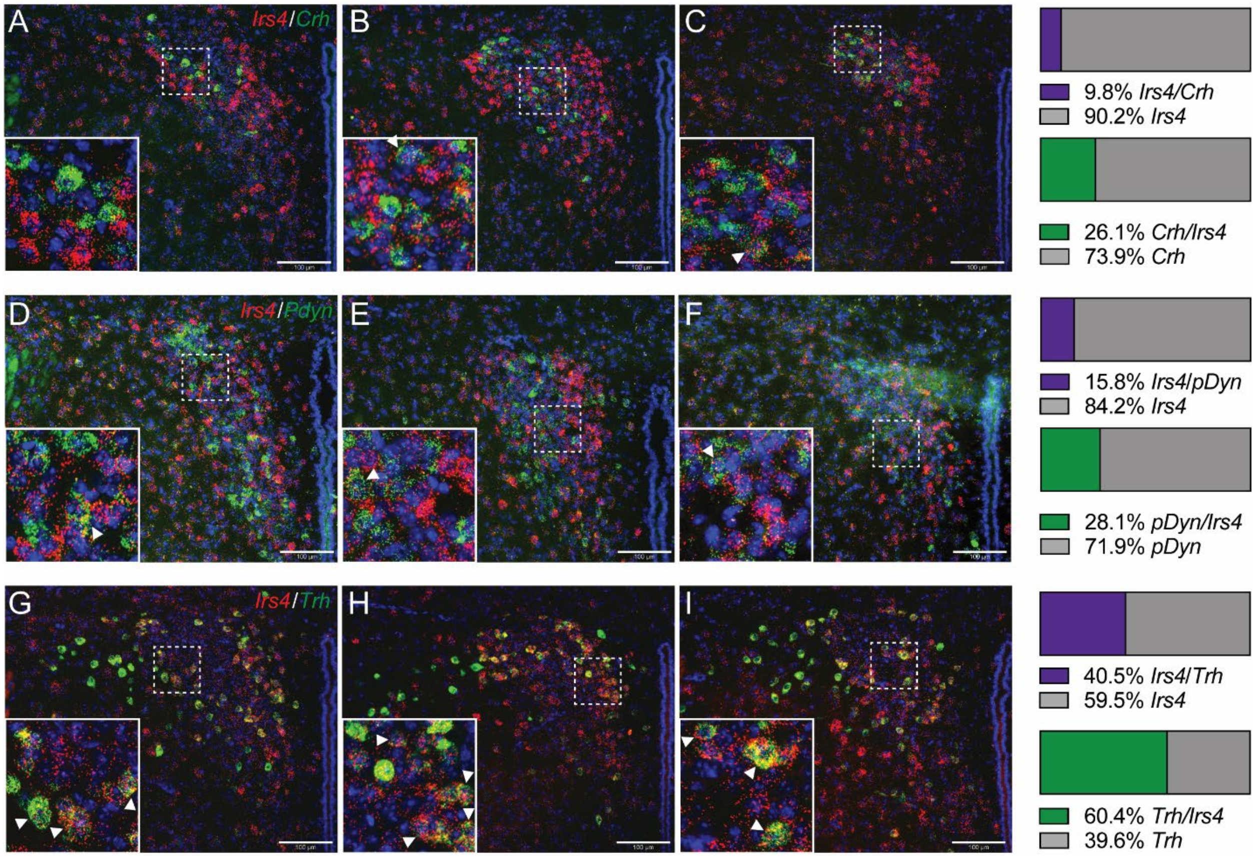

Supplementary Figure 1.1: Overlap of IRS4 with CRH, pDYN, and TRH in the PVH. Fluorescent *in situ* hybridization images demonstrate *Irs4* expression (red) overlaid with *Crh* (A-C, green), *Pdyn* (D-E, green), or *Trh* (G-I, green) and DAPI throughout. Co-expression of *Irs4* (purple) with *Crh* (A-C, right), *Pdyn* (D-F, right), or *Trh* (G-I, right), whereas the mean percentage of cells expressing *Crh*, *Pdyn*, or *Trh* that were *Irs4*$^{+}$ are represented in green (n=3). Dashed boxes represent insets that were digitally enlarged in bottom left of images. White arrowheads point to colocalized cells within insets. Scale bars represent 100 µm.

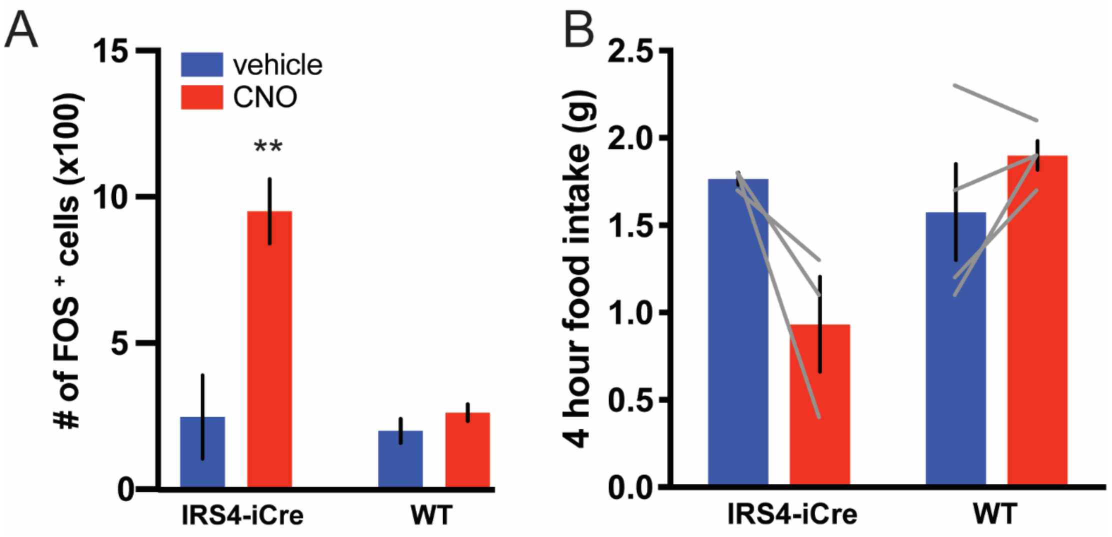

Supplementary Figure 2.1: Activation of IRS4^{PVH} neurons requires hM3Dq expression and CNO administration. Quantification of unilateral FOS induction demonstrates increased FOS in CNO-injected *IRS4-iCre* mice with Flex-hM3Dq expression compared to those injected with vehicle (p=0.006), whereas WT controls had no significant change in nuclear FOS with CNO administration. (A). Differences in four-hour (B) feeding is not observed between WT mice injected with vehicle or CNO at the onset of the dark cycle (p=0.2255, t=1.522), whereas a trend exists for decreased food intake in *IRS4-iCre* mice with Flex-hM3Dq expression in the PVH (p=0.1066, t=2.813). Average values ± SEM are shown for food intake values, average values ± SD are shown for FOS quantification. Significance was determined using a paired-t-test in comparison to vehicle values for food intake (WT n=4, *IRS4-iCre* n=3), and a one-way ANOVA followed by Tukey's post-hoc for FOS quantification (n=2/group).

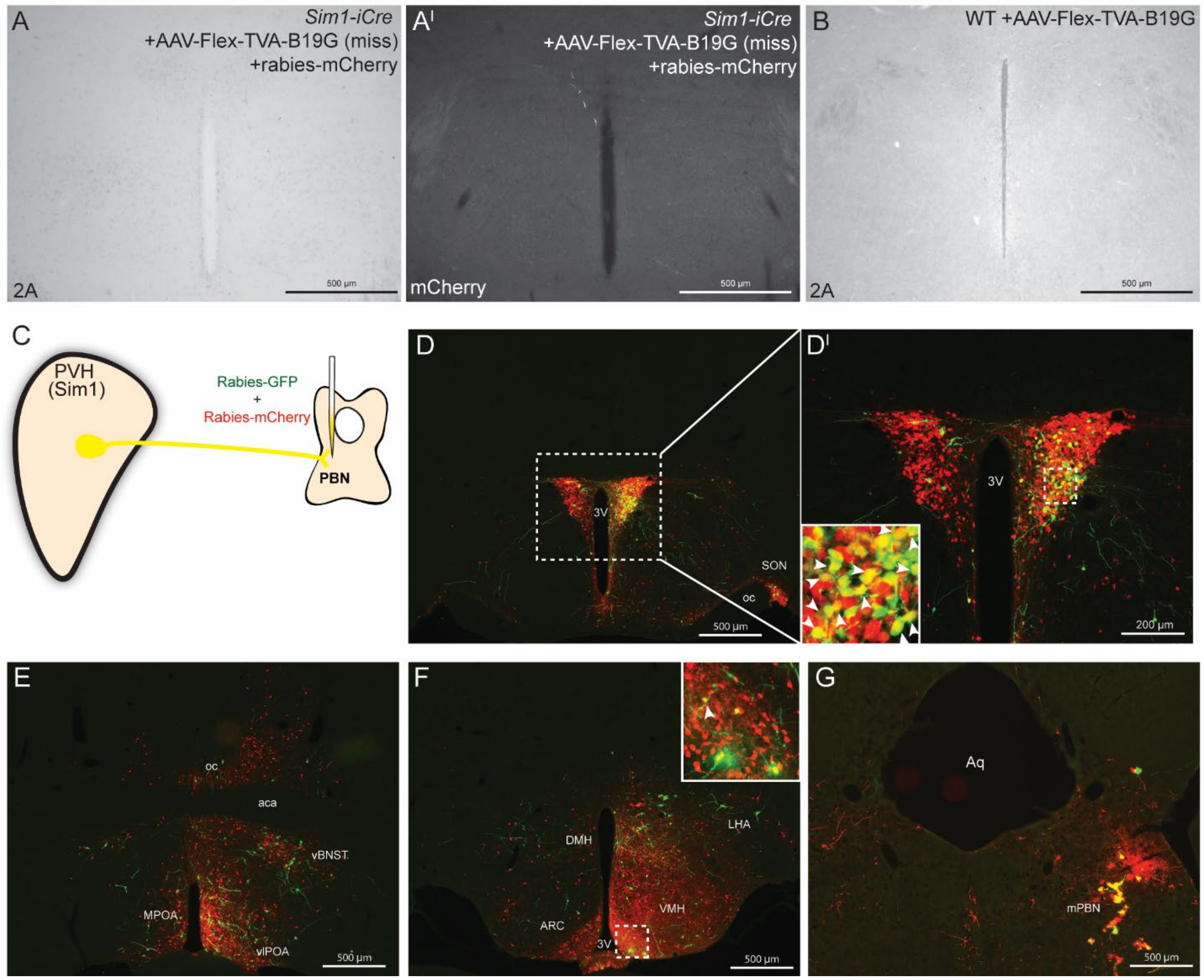

Supplementary Figure 4.1: Monosynaptic rabies virus tracing is Cre-dependent, dependent on helper virus expression, and capable of dual rabies infection. IHC in the PVH of *Sim1-Cre +AAV-Flex-TVA-B19G* mice validates a "miss" since 2A staining (reflective of AAV-Flex-TVA-B19G expression) is not detected (A). Still, NTS-directed rabies-mCherry injection in the same mouse displays limited mCherry expression in the PVH, therefore confirming the requirement of AAV-Flex-TVA-B19G expression for modified rabies virus expression (A^{I}). B) 2A expression in wildtype mice injected with AAV-Flex-TVA-B19G demonstrates that Cre recombinase is required for helper virus expression. C) Injection of both rabies-mCherry and rabies-GFP in the same PBN projection target in *Sim1-Cre +AAV-Flex-TVA-B19G* mice illustrates that both rabies viruses can be expressed in PBN-projecting PVH neurons (D). Sites upstream including the bed nucleus of the stria terminalis (BNST, E), preoptic area (POA), arcuate nucleus (ARC, F) and VMH (F) show limited co-labeling. G) Gliosis in the PBN due to rabies-mCherry/rabies-GFP injection demonstrates that both rabies-GFP and rabies-mCherry were mixed and injected at the same site. Dashed boxes indicate regions that are digitally enlarged and shown as insets. White arrowheads indicate co-localization in neurons with both rabies-GFP and rabies-mCherry. 3V=third ventricle, aca= anterior part of anterior commissure, DMH=dorsomedial hypothalamus, oc=optic chiasm, LHA=lateral hypothalamic area.

	PVH^{TetTox}	Unil. PVH^{TetTox}	PVH^{Flex}	WT^{TetTox}
BW (g, t=0)	27.17 ±2.02	24.78 ±0.60	25.97 ±0.69	23.78 ±1.01
BW (g, t=8wk)	36.80 ±4.02***†	31.64 ±1.73	30.58 ±0.72	26.54 ±0.71
Change in BW (g, t=8wk)	10.50 ±1.92***††	7.14 ±1.46	4.71 ±0.66	2.96 ±0.75
Early weekly food intake (g)	39.27 ±3.78*	36.42 ±2.36	35.90 ±1.24	30.03 ±1.46
Late weekly food intake (g)	33.55 ±1.35	30.30 ±4.11	32.30 ±2.36	27.81 ±1.67
VO_2 (ml/kg LBM)	4540 ±167.0	4294 ±194.0	4255 ±170.2	4477 ±191.8
Average total X activity (counts/hr)	1106 ±44.98	1392 ±37.84	1011 ±49.13	1194 ±140.3
body fat (%, early)	19.21 ±1.24**††	12.89 ±0.98§	12.90 ±1.09	13.05 ±0.89
body fat (%, late)	21.86 ±1.85**†	12.66 ±1.83§§	15.17 ±1.08	13.38 ±0.861

Supp. Table 5.1. Unilateral $IRS4^{PVH}$ silencing alters bodyweight physiology. Comparison of bodyweight and CLAMS results from $IRS4^{PVH}$ TetTox cohorts with viral injections that only targeted one side of the PVH (Unil. PVH^{TetTox}) still demonstrate trends toward changes in physiology. Data represented includes results illustrated in Fig. 5. Data was analyzed using a one-way ANOVA followed by Tukey's multiple comparisons if appropriate, or an unpaired t-test (number of cells infected with AAV-Flex-TetTox between PVH^{TetTox} and Unil. PVH^{TetTox} cohorts). Average values ± SEM are shown. *p<0.05, ***p<0.001 for PVH^{TetTox} compared to WT^{TetTox}; †p<0.05 for PVH^{TetTox} compared to PVH^{Flex}; §p<0.05 for PVH^{TetTox} compared to Unil. PVH^{TetTox}; &p<0.05 for PVH^{Flex} compared to WT^{TetTox}; $p<0.05 for Unil. PVH^{TetTox} compared to WT^{TetTox}. PVH^{TetTox} n=6 (BW, t=0; early food intake, CLAMS, early body composition), n=5 (BW, t=8wk, change in BW, late body composition), n=4 (late food intake); Unil. PVH^{TetTox} n=6 (BW t=0wk, early food intake), n=5 (BW t=8wk, change in BW, late food intake), n=4 (CLAMS, body composition); WT^{TetTox} n=13 (BW t=0wk, early food intake, CLAMS), n=11 (BW t=8wk, change in BW, body composition), n=9 (late food intake); PVH^{Flex} n=17 (BW t=0, early food intake, late body composition), n=16 (BW t=8wk, change in BW), n=8 (late food intake, CLAMS), n=10 (early body composition).
